# Measuring the health-related quality of life of children with impaired mobility: examining correlation and agreement between children and parent proxies

**DOI:** 10.1186/s13104-017-2683-9

**Published:** 2017-08-10

**Authors:** Nathan Bray, Jane Noyes, Nigel Harris, Rhiannon Tudor Edwards

**Affiliations:** 1 Centre for Health Economics and Medicines Evaluation, Bangor University, Ardudwy, Normal Site, Bangor, Gwynedd LL57 2PZ UK; 2 School of Social Sciences, Bangor University, Neuadd Ogwen, Bangor, Gwynedd LL57 2DG UK; 3 DesignAbility, Bath Institute of Medical Engineering, The Wolfson Centre, Royal United Hospital, Bath, BA1 3NG UK

**Keywords:** Mobility impairment, Childhood disability, Wheelchair, Assistive technology, Health economics, Health-related quality of life

## Abstract

**Objective:**

The objective of this research project was to evaluate the validity of proxy health-related quality of life measures in the context of paediatric mobility impairment. Accurate health-related quality of life data is essential for quality-adjusted life year calculation; a key outcome in economic evaluation. Thirteen child-parent dyads (13 children with mobility impairments, 13 parent proxies) were asked to complete a range of outcome measures (EQ-5D-Y, VAS and HUI2/3) relating to the child’s health. The relationship between respondent outcomes was examined using tests of respondent type effect (Wilcoxon signed-rank), correlation (Spearman’s rank-order) and agreement (Bland–Altman plots).

**Results:**

Parent proxies significantly undervalued the health-related quality of life of their mobility-impaired children: children rated their health-related quality of life higher than their parents by proxy on all measures. The VAS had the highest overall mean score for children and proxies (79.50 [SD = 15.01] and 75.77 [SD = 14.70] respectively). Child and proxy results were significantly different (p < 0.05) for all measures besides the VAS (p = 0.138). Strong correlation and acceptable agreement were observed for equivalent child/proxy VAS and HUI measures. The EQ-5D-Y exhibited the least agreement between children and proxies. Sufficient association between child/proxy VAS and HUI measures indicated a degree of interchangeability.

## Introduction

Preference-based measures of health-related quality of life (HRQoL) are used to assess the utility gains associated with clinical, social care and public health interventions. Utility refers to the subjective level of wellbeing experienced in different states of health [1]. Each potential health state is assigned a utility weight derived from the social desirability of that state, for instance ranging from death to perfect health [2]. The most commonly used measure of utility is the quality adjusted life-year (QALY), which is an aggregate of both quantity and quality of life. In order to calculate QALYs preference-based HRQoL data is required.

HRQoL is a subjective and multi-dimensional construct defined as the perceived impact of health status on quality of life, including physical, psychological and social functioning [3]. Definitions of paediatric HRQoL must take into account the unique social contexts of childhood, including family, friends and school [4].

Standard measures of HRQoL, such as the EQ-5D [5] and Health Utilities Index (HUI) [6], are supported by a wealth of validation literature and value sets to ascribe weights to utility functions [5–10].

In paediatric research it is common place for parents and/or carers to report outcomes on behalf of their child when age, ability or capacity precludes inclusion of the child directly. However, proxy reports are often significantly different to self-reports, particularly for children with disabilities [11, 12]. Understanding the relationship between child self-reported data and parental proxy data is important to assess the relative validity of different sources of data in economic analyses [13].

Previous research has demonstrated the holistic benefits of wheelchairs for children, however there is practically no evidence to demonstrate the relative cost-effectiveness of paediatric wheelchair provision [14]. To facilitate future economic evaluations in this context, there is a need to understand how best to measure the HRQoL of children with mobility impairments.

The overarching aim of this pilot study was to compare how children with mobility impairments and their parents (by proxy) report HRQoL using standard outcome measures. Our secondary objective was to determine if there are statistically significant differences between the self-reported outcomes of children with mobility impairments and the proxy-reported outcomes of their parents.

Data were collected as part of a Ph.D. studentship programme of research. Other findings from the project have been published elsewhere [14–16].

## Methods

### Sampling and recruitment

Inclusion and exclusion criteria are presented in Table 1. The sampling frame comprised dyads of children with mobility impairments (aged 18 or under; hereafter referred to as ‘children’) and one of their parents. Participants were offered a small financial incentive (a £10 retail voucher) for taking part in the study. Children under the age of 16 completed an assent form and their parents completed a proxy consent form. A sample size calculation was not undertaken due to the small scale of the pilot.

**Table 1:** Inclusion and exclusion criteria for participants

Inclusion criteria	Exclusion criteria
Children and young people with long term (>6 months^a^) mobility impairments Aged 5–18 years Requires a manual and/or powered wheelchair/pushchair/buggy for the purposes of mobility Able to give informed consent to take part in study, or able to give assent and parent/guardian able to give proxy consent Parent(s) or legal guardian(s) of a child or young person with a long term mobility impairment who uses a wheelchair Able to give informed consent to take part in study, and able to give proxy consent where required	Any significant social or emotional problems or challenging behaviours where such problems in the opinion of the family or clinical team are likely to impair participant’s ability to take part in the study or pose a risk to the researcher or the participant Unable to communicate in English or Welsh

^a^ Long term mobility impairment defined as having existed for 6 months or more, or expected to last for 6 months or more

Participants were recruited between June and October 2013 from two UK recruitment sites: a Welsh National Health Service (NHS) wheelchair service and a children’s wheelchair charity based in England.

Data were collected using postal questionnaire surveys. Questionnaires contained outcome measures (EQ-5D-Y, HUI, VAS) and demographic questions. Child questionnaires contained self-administered versions of measures, while parent questionnaires contained proxy versions.

### Measures

The EQ-5D-Y is a validated HRQoL measure for use in children and parent proxies [17]. A pre-existing UK general adult population value set was used to assign weights for domain levels, on a death to perfect health scale (0 to 1) [7]. At present there are no specific value sets for children or parent proxies.

The HUI is a validated HRQoL measure containing the HUI2 and HUI3 systems [6]. Multi-attribute utility functions were used to assign utility scores to HUI2/3 attribute levels, on a death to perfect health scale (0 to 1) [9, 10]. Both the EQ-5D-Y and HUI measures have health states considered worse than death, and thus some states can have negative values.

The VAS (EQ-VAS) is typically presented alongside the EQ-5D-Y, and measures self-rated health status on a scale from worst imaginable to best imaginable health (0 to 100). In order to aid comparison with the EQ-5D-Y and HUI measures the VAS scoring system was converted from a 0 to 100 scale to a 0 to 1 scale during certain analyses.

### Analyses

*Statistical analysis of mean scores* All analyses were conducted using SPPS v20. Data were not normally distributed therefore non-parametric statistical methods were used. Wilcoxon signed-rank tests were used to analyse statistically significant (p < 0.05) differences between children and parent proxies, based on EQ-5D-Y, VAS and HUI total score mean ranks for dyads.

*Correlation between children and parent proxies* Spearman’s rank-order was used to test correlation. Correlation was assessed between dyads of child and parent proxy total scores to examine whether they were associated. In order for child and parent proxy measures to be considered sufficiently associated, correlation coefficients had to be defined as moderate or strong (*r*_*s*_ of <0.20 = absent; *r*_*s*_ of 0.20 to 0.35 = weak; *r*_*s*_ of 0.35 to 0.50 = moderate; *r*_*s*_ of ≥0.50 = strong) [18].

*Agreement between children and parent proxies* Bland–Altman plots were used to assess agreement between children and parent proxy results. On the plot 95% of differences should lie between the established limits of agreement (mean difference ± 1.96 SD) [19], represented as dashed lines. Where bias or limits of agreement are beyond those deemed acceptable for clinical use, the measures lack agreement to be used interchangeably to measure the same construct. A confidence limit of 0.50 was chosen.

## Results

### Response rate, sample size and missing data

Study invitation packs were sent to 64 eligible children and their parents. In total 28 questionnaires were returned; 15 child participants and 13 parent proxies. Two child participants returned questionnaires without parent proxy data, and thus were excluded from the dyad analyses. This provided a full sample of 13 dyads of children and their parents. Data from two dyads were excluded from the EQ-5D-Y analyses due to incompletion of the measure, giving an overall completion rate of 84.6% for this measure. All other measures were completed in full without error or missing data. Demographic details for the dyads of children and parents are presented in Table 2.

**Table 2:** Demographic characteristics of dyads of children with mobility impairments and parents

Demographic characteristics	Number (%)
Study site	
NHS Wheelchair Service	2 (15.4)
Wheelchair charity	11 (84.6)
Parent gender	
Female	12 (92.3)
Male	1 (7.7)
Parent age	
30–39 years	3 (23.1)
40–49 years	8 (61.5)
50–59 years	2 (15.4)
Parent ethnicity	
White British	13 (100)
Parent education	
Higher	4 (30.7)
Further (e.g. a level)	2 (15.4)
GCSE/O level	2 (15.4)
Other	3 (23.1)
None	2 (15.4)
Annual household income	
£5000–£15,000	1 (7.7)
£16,000–£25,000	1 (7.7)
£26,000–£35,000	1 (7.7)
£36,000–£50,000	6 (46.2)
£51,000–£75,000	2 (15.4)
£75,000 or more	1 (7.7)
Missing	1 (7.7)
Parent employment status	
Full-time	1 (7.7)
Part-time	6 (46.2)
Unemployed	6 (46.2)
Child’s condition	
Cerebral palsy	11 (84.6)
Hemiplegia/stroke	1 (7.7)
Muscular dystrophy	1 (7.7)
Child age	
6–15 years	7 (58.8)
16–18 years	6 (46.2)
Child gender	
Female	5 (38.5)
Male	8 (61.5)
Child education	
Primary school	2 (15.4)
High school	5 (38.5)
College	4 (30.7)
University	1 (7.7)
Home schooled	1 (7.7)
Frequency of child’s equipment use	
Most of the time	2 (15.4)
All of the time	11 (84.6)
Type of equipment used by child	
Manual	4 (33.3)
Manual and EPIOC	9 (66.7)

% refers to the percentage of research participants

### HRQoL total score results

**Fig. 1:**
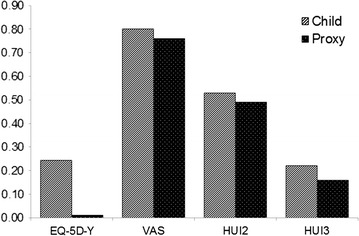
Mean EQ-5D-Y, VAS, HUI2 and HUI3 total scores for children with mobility impairments and parent proxies. Figure showing mean EQ-5D-Y, VAS, HUI2 and HUI3 total scores for children with mobility impairments and parent proxies. The EQ-5D-Y and HUI measures are scored on a 0 to 1 scale, while the VAS is scored on a 0 to 100 scale. For the purpose of this comparison, the VAS has been converted to a 0 to 1 scale

Descriptive statistics are presented in Table 3. The overall mean scores on all of the measures were higher for child self-reports than for parent proxies (see Fig. 1). The VAS had the highest overall mean score for children and parent proxies (79.50 [SD = 15.01] and 75.77 [SD = 14.70] respectively), followed by the HUI2 (0.53 [SD = 0.07] and 0.49 [SD = 0.09] respectively). Children scored the EQ-5D-Y higher than the HUI3 (0.24 [SD = 0.30] and 0.22 [SD = 0.09] respectively), while parent proxies scored the EQ-5D-Y lower than the HUI3 (0.01 [SD = 0.14] and 0.16 [SD = 0.10] respectively). All scores were below child population norms: 0.89 for EQ-5D-Y [20]; 83.17 for VAS [20]; 0.95 for HUI2 [21, 22]; and 0.85 to 0.92 for HUI3 [8].

**Table 3:** Outcome measure results and descriptive statistics (by child age group) for children with mobility impairments and parent proxies

Age	Child self-report			Parent proxy		
	6–15	16–18	All	6–15	16–18	All
EQ-5D-Y^a^						
Mean	0.08	0.52	0.24	0.00	0.04	0.01
SD	0.14	0.30	0.30	0.18	0.04	0.14
Median	0.03	0.65	0.23	0.03	0.04	0.03
25th	−0.02	0.46	0.02	−0.05	0.00	0.00
75th	0.23	0.71	0.41	0.10	0.07	0.07
N	7	4	11	7	4	11
VAS^b^						
Mean	84.29	74.00	79.54	77.86	73.33	75.77
SD	15.92	12.98	15.01	15.51	14.72	14.70
Median	90.00	78.50	81.00	80.00	75.00	80.00
25th	85.00	71.75	77.00	77.50	66.25	70.00
75th	91.00	80.00	90.00	87.50	83.75	85.00
N	7	6	13	7	6	13
HUI2^c^						
Mean	0.52	0.56	0.53	0.46	0.52	0.49
SD	0.08	0.07	0.07	0.12	0.06	0.09
Median	0.53	0.55	0.54	0.43	0.54	0.46
25th	0.45	0.54	0.46	0.41	0.48	0.43
75th	0.55	0.59	0.55	0.48	0.56	0.54
N	7	6	13	7	6	13
HUI3^d^						
Mean	0.20	0.24	0.22	0.14	0.18	0.16
SD	0.09	0.10	0.09	0.13	0.06	0.10
Median	0.22	0.24	0.22	0.10	0.21	0.16
25th	0.16	0.21	0.21	0.06	0.17	0.07
75th	0.25	0.29	0.27	0.19	0.22	0.22
N	7	6	13	7	6	13

^a^ −0.594 = minimum value, 1 = maximum value (0 = death; 1 = perfect health)

^b^ 0 = minimum value, 100 = maximum value (0 = worst possible health; 1 = best possible health)

^c^ −0.03 = minimum value, 1 = maximum value (0 = death; 1 = perfect health)

^d^ −0.36 = minimum value, 1 = maximum value (0 = death; 1 = perfect health)

### Statistical analysis of child and parent proxy dyads HRQoL scores

A significant effect of respondent type was found for all measures besides the VAS. Child self-reported total scores were significantly higher for the EQ-5D-Y (*Z* = −2.525, *p* = 0.012), HUI2 (*Z* = −2.310, *p* = 0.021) and HUI3 (*Z* = −2.599, *p* = 0.009). Mean child VAS scores were higher, but not significantly (*Z* = −1.483, *p* = 0.138). See Table 3 for median score results.

### Correlation between child self-report and parent proxy measures

Correlation coefficients are presented in Table 4. Significant strong (*p* < 0.05) correlations were found between dyads of child and parent proxy results for the EQ-5D-Y (*r*_*s*_ = 0.665, *p* = 0.026), HUI2 (*r*_*s*_ = 0.728, *p* = 0.005) and HUI3 (*r*_*s*_ = 0.842, *p* < 0.001). Strong significant correlation was also found between the child HUI3 and parent proxy HUI2 (*r*_*s*_ = 0.567, *p* = 0.043); the child HUI2 and parent proxy HUI3 (*r*_*s*_ = 0.932, *p* < 0.001); and the child EQ-5D-Y and parent proxy HUI2 (*r*_*s*_ = 0.637, *p* = 0.039). A strong but non-significant correlation was found between the child and parent proxy VAS (*r*_*s*_ = 0.545, *p* = 0.054). Therefore, convergence between equivalent child and parent proxy measures was sufficient, with only the parent HUI3 and child HUI2 exhibiting stronger correlations with non-equivalent measures.

**Table 4:** Correlations between child self-reported and parent proxy results

	Child EQ-5D-Y	Child VAS	Child HUI2	Child HUI3
Parent EQ-5D-Y	0.665*	−0.177	0.279	−0.167
Parent VAS	0.075	0.545	−0.187	−0.298
Parent HUI2	0.627*	−0.329	0.728*	0.567*
Parent HUI3	0.290	−0.537	0.932*	0.842*

* Significant correlation at 0.05 level (2-tailed)

Strength of correlation: <0.20 = absent; 0.20 to 0.35 = weak; 0.35 to 0.50 = moderate; ≥0.50 = strong

### Agreement between child self-report and parent proxy measures (dyads)

**Fig. 2:**
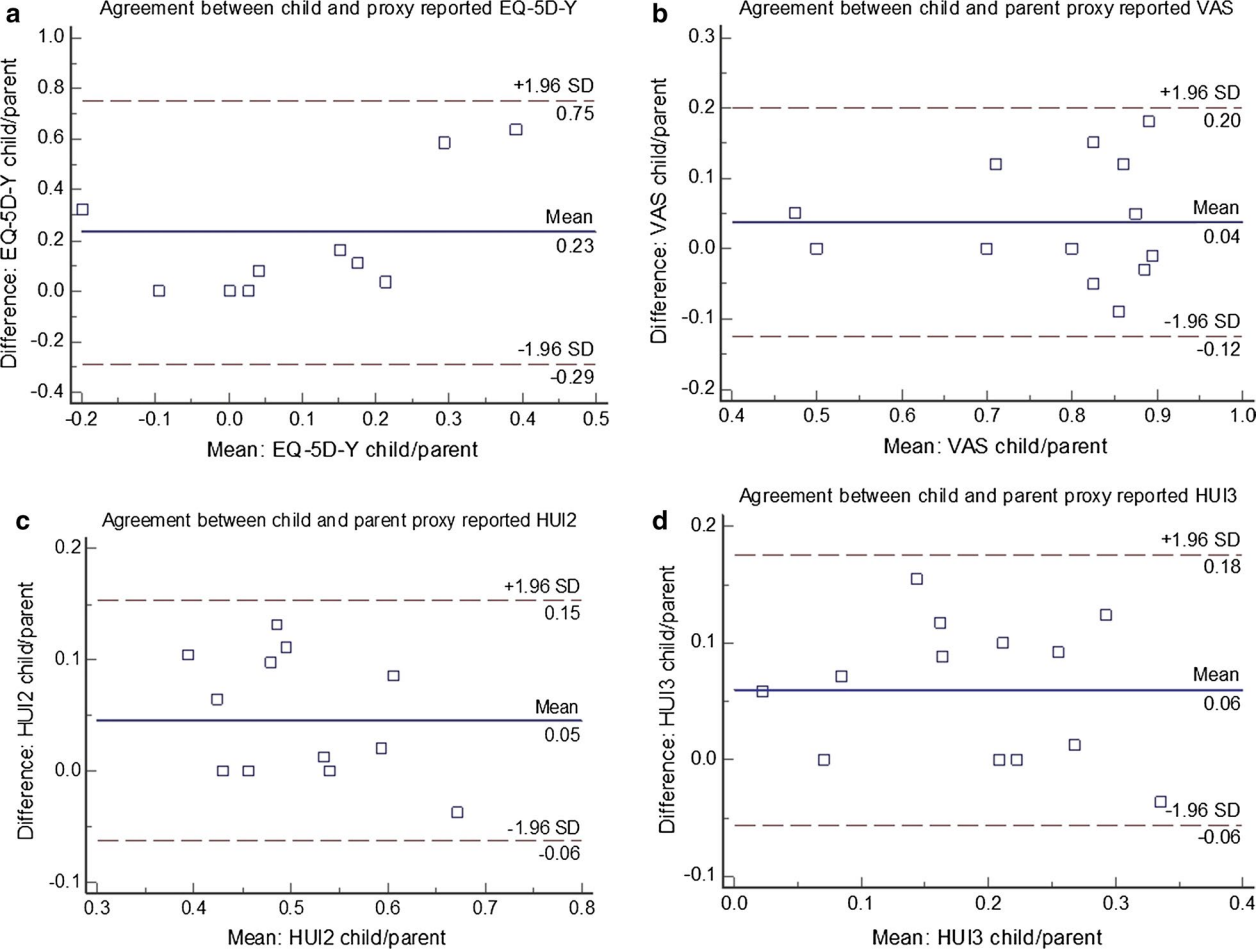
Bland–Altman plots: agreement between children with mobility impairments and parent proxies for EQ-5D-Y **a**, VAS **b**, HUI2 **c** and HUI3 **d**. On the plots 95% of differences should lie between the established limits of agreement (mean difference ± 1.96 SD), represented as *dashed lines*. Where bias or limits of agreement are beyond those deemed acceptable for clinical use, the measures lack agreement to be used interchangeably to measure the same construct. The EQ-5D-Y and HUI measures are scored on a 0 to 1 scale, while the VAS is scored on a 0 to 100. For the purpose of this comparison, the VAS has been converted to a 0 to 1 scale

Sufficient agreement was found between the child and parent proxy HUI2 (*confidence limit* [*CL*] = 0.22), HUI3 (*CL* = 0.22) and VAS measures (*CL* = 0.32) (see Table 5; Fig. 2), with the HUI measures showing the most agreement between child and parent scores. The EQ-5D-Y exhibited clinically important discrepancies between child and parent proxy responses (*CL* = 1.04) thus showing insufficient agreement to be used interchangeably in this cohort.

**Table 5:** Comparing mobility impaired child self-reported and parent proxy outcomes: agreement, correlation and respondent type effect

	N (Dyads)	Mean difference	95% confidence limits	Overall agreement limit	Wilcoxon signed rank *p*	Spearman’s Rho correlation coefficient^b^
EQ-5D-Y	11	0.24	−0.29 to 0.75	1.04	0.012*	0.665*
VAS^a^	13	0.04	−0.12 to 0.20	0.32	0.138	0.545
HUI2	13	0.05	−0.06 to 0.15	0.22	0.021*	0.728*
HUI3	13	0.16	−0.06 to 0.18	0.22	0.009*	0.842*

* Significant at 0.05 level

^a^ Converted to 0 to 1 scale

^b^ Strength of correlation: <0.20 = absent; 0.20 to 0.35 = weak; 0.35 to 0.50 = moderate; ≥0.50 = strong

## Discussion

The EQ-5D-Y did not sufficiently measure HRQoL in accordance with how the sampled children and parents defined overall health status. This is likely due to general population health state preferences being unrepresentative of how children with mobility impairments (and their parents by proxy) value their own health state.

The simple descriptive system of the EQ-5D-Y lacks nuance for children with mobility impairments. For instance, the EQ-5D-Y mobility domain has no consideration for mobility beyond walking and thus automatically discounts the HRQoL of a mobility impaired child, even though they may be mobile using assistive technology solutions. The relationship between gross motor function is often contradictory [23], thus measures such as the EQ-5D and HUI lack sufficient sensitivity to be used appropriately in mobility impaired populations.

VAS mean scores were to a large extent higher than the other measures in this study, which raises questions about the validity of the HRQoL measures, in particular the value sets used, as the VAS is a clear indicator of self and proxy reported health status. Future research into child-specific value sets could help to improve sensitivity of HRQoL measures, however it is currently unclear whether young children can adequately value health states, or whether it is appropriate to use preference weights from wider society or proxy reports [24].

Previous research has similarly demonstrated the issue of using proxy HRQoL data, as proxy scores are often systematically lower than self-reports of children with disabilities [11, 12]. In practice child self-reported data should be prioritised over proxy data. In circumstances where this is not possible, the VAS appears to be more valid than the EQ-5D-Y and HUI2/3 for measuring health outcomes of children with mobility impairments. Further research should focus on establishing whether systematic differences between child and proxy reports can be sufficiently predicted and used to adjust proxy outcomes.

### Methodological considerations

Significant differences were found between child and parent proxy measures, and yet they were also correlated. These two concepts would appear to be mutually exclusive, but in fact demonstrate the issue with using just statistical analysis of respondent type effect or correlation to assess relationships between measures. Statistically significant differences in mean scores demonstrate that mean scores are sufficiently different to be significant, however this does not indicate the relationship between scores across the cohort. Correlation is an indication of association, for instance as child scores goes up so do parent scores. This, however, does not give an indication as to whether these scores are in agreement; a large difference between child and parent scores may exist but they may also vary in a similar manner, and thus still be correlated. Tests of agreement should supplement analyses of correlation in order to gain a better understanding of the relationship between measures and/or respondents.

### Limitations

The size of the sample was small and thus the analyses lacked power. Furthermore, the demographic characteristics were not representative of the wider population of children with mobility impairments in the UK. Dyads with missing data were excluded from analyses, which reduced the sample size. In future research a larger sampling frame is required. Comparing the results of the VAS with validated HRQoL measures raises some issues, as the VAS is not preference-based and thus does not provide an indication of the relative societal value of different states of health. Therefore, VAS results are not directly comparable with more complex preference-based measures. EuroQoL do not recommend the use of the adult EQ-5D value set for scoring the EQ-5D-Y. Despite these shortcomings, this pilot demonstrates that children with mobility impairments can assess their own HRQoL and health status, thus their views should be prioritised in outcome measurement.

## Abbreviations

CHU-9D: child health utility nine dimension
CL: confidence limit
EPIOC: electrically powered indoor/outdoor chair
EQ-5D: EuroQoL five dimension
EQ-5D-Y: EuroQoL five dimension youth version
HRQoL: health-related quality of life
HUI: health utilities index
NHS: National Health Service
NICE: National Institute for Health and Care Excellence
QALY: quality-adjusted life year
SD: standard deviation
UK: United Kingdom
VAS: visual analogue scale

## References

[1] Robinson R (1993). Cost-utility analysis. BMJ.

[2] Stevens K, Palfreyman S (2012). The use of qualitative methods in developing the descriptive systems of preference-based measures of health-related quality of life for use in economic evaluation. Value Health.

[3] Leidy NK, Rich M, Geneste B (1999). Recommendations for evaluating the validity of quality of life claims for labelling and promotion. Value Health.

[4] Matza LS, Swensen AR, Flood EM, Secnik K, Leidy NK (2004). Assessment of health-related quality of life in children: a review of conceptual, methodological, and regulatory issues. Value Health.

[5] EuroQol Group (1990). EuroQol-a new facility for the measurement of health-related quality of life. Health Policy.

[6] Horsman J, Furlong W, Feeny D, Torrance G (2003). The Health Utilities Index (HUI): concepts, measurement properties and applications. Health Qual Life Outcomes.

[7] Dolan P, Gudex C, Kind P, Williams A (1996). The time trade-off method: results from a general population study. Health Econ.

[8] Pogany L, Barr RD, Shaw A, Speechley KN, Barrera M, Maunsell E (2006). Health status in survivors of cancer in childhood and adolescence. Qual Life Res.

[9] Torrance GW, Feeny DH, Furlong WJ, Barr RD, Zhang Y, Wang Q (1996). Multiattribute utility function for a comprehensive health status classification system. Health Utilities Index Mark 2. Med Care.

[10] Feeny DH, Furlong WJ, Torrance GW, Goldsmith CH, Zhu Z, DePauw S, Denton M, Boyle M (2002). Multi-attribute and single-attribute utility functions for the health utilities index mark 3 system. Med Care.

[11] Varni JW, Burwinkle TM, Sherman SA, Hanna K, Berrin SJ, Malcarne VL, Chambers HG (2007). Health-related quality of life of children and adolescents with cerebral palsy: hearing the voices of the children. Dev Med Child Neurol.

[12] Bray P, Bundy AC, Ryan MM, North KN, Everett A (2010). Health-related quality of life in boys with Duchenne muscular dystrophy: agreement between parents and their sons. J Child Neurol.

[13] Eiser C, Morse R (2001). Quality-of-life measures in chronic diseases of childhood. Health Technol Assess.

[14] Bray N, Noyes J, Edwards RT, Harris N (2014). Wheelchair interventions, services and provision for disabled children: a mixed-method systematic review and conceptual framework. BMC Health Serv Res.

[15] Bray N, Yeo ST, Noyes J, Harris N, Edwards RT (2016). Prioritising wheelchair services for children: a pilot discrete choice experiment to understand how child wheelchair users and their parents prioritise different attributes of wheelchair services. Pilot Feasibility Stud..

[16] Bray N, Noyes J, Harris N, Edwards RT (2017). Defining health-related quality of life for young wheelchair users: a qualitative health economics study. PLoS ONE.

[17] van Reenen M, Janssen B, Oppe M, Kreimeier S, Greiner W (2014). EQ-5D-Y user guide: basic information on how to use the EQ-5D-Y instrument.

[18] Juniper EF, Guyatt GH, Jaeshke R, Spiker B (1996). How to develop and validate a new health-related quality of life instrument. Quality of life and pharmacoeconomics in clinical trials.

[19] Bland JM, Altman DG (1986). Statistical methods for assessing agreement between two methods of clinical measurement. Lancet.

[20] Noyes J (2004). Evaluation of health and social provision for ventilator dependent children in the UK: costs and outcomes [dissertation].

[21] Saigal S, Feeny D, Furlong W, Rosenbaum P, Burrows E, Torrance G (1994). Comparison of the health-related quality of life of extremely low birth weight children and a reference group of children at age eight years. J Pediatr.

[22] Feeny DH, Furlong W, Saigal S, Sun J (2004). Comparing directly measured standard gamble scores to HUI2 and HUI3 utility scores: group- and individual-level comparisons. Soc Sci Med.

[23] Livingston MH, Rosenbaum PL, Russell DJ, Palisano RJ (2007). Quality of life among adolescents with cerebral palsy: what the literature tell us?. Dev Med Child Neurol.

[24] Wille N, Badia X, Bonsel G, Burström K, Cavrini G, Devlin N, Egmar A-N, Greiner W, Gusi N, Herdman M, Jelsma J, Kind P, Scalone L, Ravens-Sieberer U (2010). Development of the EQ-5D-Y: a child-friendly version of the EQ-5D. Qual Life Res.

